# A Novel Microbiosensor Microarray for Continuous *ex Vivo* Monitoring of Gamma-Aminobutyric Acid in Real-Time

**DOI:** 10.3389/fnins.2018.00500

**Published:** 2018-08-07

**Authors:** Imran Hossain, Chao Tan, Phillip T. Doughty, Gaurab Dutta, Teresa A. Murray, Shabnam Siddiqui, Leonidas Iasemidis, Prabhu U. Arumugam

**Affiliations:** ^1^Institute for Micromanufacturing, Louisiana Tech University, Ruston, LA, United States; ^2^Center for Biomedical Engineering and Rehabilitation Science, Louisiana Tech University, Ruston, LA, United States

**Keywords:** electrochemical, GABA, glutamate, biosensor, microarray, neurochemical, *ex vivo*

## Abstract

Gamma-aminobutyric acid (GABA) is a major inhibitory neurotransmitter that is essential for normal brain function. It is involved in multiple neuronal activities, including plasticity, information processing, and network synchronization. Abnormal GABA levels result in severe brain disorders and therefore GABA has been the target of a wide range of drug therapeutics. GABA being non-electroactive is challenging to detect in real-time. To date, GABA is detected mainly via microdialysis with a high-performance liquid chromatography (HPLC) system that employs electrochemical (EC) and spectroscopic methodology. However, these systems are bulky and unsuitable for real-time continuous monitoring. As opposed to microdialysis, biosensors are easy to miniaturize and are highly suitable for *in vivo* studies; they selectively oxidize GABA into a secondary electroactive product (usually hydrogen peroxide, H_2_O_2_) in the presence of enzymes, which is then detected by amperometry. Unfortunately, this method requires a rather cumbersome process with prereactors and relies on externally applied reagents. Here, we report the design and implementation of a GABA microarray probe that operates on a newly conceived principle. It consists of two microbiosensors, one for glutamate (Glu) and one for GABA detection, modified with glutamate oxidase and GABASE enzymes, respectively. By simultaneously measuring and subtracting the H_2_O_2_ oxidation currents generated from these microbiosensors, GABA and Glu can be detected continuously in real-time *in vitro* and *ex vivo* and without the addition of any externally applied reagents. The detection of GABA by this probe is based upon the *in-situ* generation of α-ketoglutarate from the Glu oxidation that takes place at the Glu microbiosensor. A GABA sensitivity of 36 ± 2.5 pA μM^-1^cm^-2^, which is 26-fold higher than reported in the literature, and a limit of detection of 2 ± 0.12 μM were achieved in an *in vitro* setting. The GABA probe was successfully tested in an adult rat brain slice preparation. These results demonstrate that the developed GABA probe constitutes a novel and powerful neuroscientific tool that could be employed in the future for *in vivo* longitudinal studies of the combined role of GABA and Glu (a major excitatory neurotransmitter) signaling in brain disorders, such as epilepsy and traumatic brain injury, as well as in preclinical trials of potential therapeutic agents for the treatment of these disorders.

## Introduction

The development of multiplexed neural probes for real-time sensing of neurochemicals is a critical step in the study and effective treatment of brain disorders. Abnormal neurochemical signaling is an underlying signature of brain dynamical disorders such as epilepsy, Parkinson’s and Alzheimer’s, traumatic brain injury, as well as drug addiction (Cahill et al., 1996; Robinson and Wightman, 2007; Robinson et al., 2008; Sandberg and Garris, 2010; Willuhn et al., 2010). Therefore, it is crucial to be able to monitor and understand the long-term spatio-temporal dynamics of key neurochemicals in the brain. Gamma-aminobutyric acid (GABA), a major inhibitory neurotransmitter, is essential for normal neuronal activity, information processing and plasticity, and for neuronal network synchronization (Caudill et al., 1982; Smith and Sharp, 1994; Bhat et al., 2010). GABA’s function is impaired in psychiatric and neurological disorders, inflammation and immune diseases, and therefore has been the target in a wide range of drug therapies (Caudill et al., 1982; Smith and Sharp, 1994; Ting Wong et al., 2003; Tian et al., 2004; Bhat et al., 2010; Auteri et al., 2015). GABA is non-electroactive, and it is therefore challenging to detect it in real-time using electrochemical (EC) and spectrophotometrical methods (Cifuentes Castro et al., 2014). To date, *in vivo* GABA levels are detected mainly via microdialysis on a high-performance liquid chromatography (HPLC) system with EC and spectroscopic detection methods (Kehr and Ungerstedt, 1988; Rowley et al., 1995b; Monge-Acuña and Fornaguera-Trías, 2009; Reinhoud et al., 2013). Since these methods are relatively insensitive to GABA, one must derivatize the solution by making it more conducive to electric signals. Several studies have used HPLC with pre/post derivatized columns using 2,4,6-trinitrobenzenesulfonic acid, o-phthaldialdehyde (OPA)-sulfite and OPA-alkylthiols to separate GABA and then detect it electrochemically at picomolar concentrations by a glassy carbon electrode (GCE) in rat brains (Caudill et al., 1982). The use of OPA-butylthiol was first proposed by Kehr, who infused nipecotic acid and 3-mercaptopro-pionic acid to obtain a faster and more sensitive determination of GABA (Kehr and Ungerstedt, 1988). Rowley et al. (1995b) extended this derivatization technique to separate seven amino acids and concluded that their method could detect, with good sensitivity, the stimulated levels of GABA and glutamate (Glu), a major excitatory neurochemical in a rat hippocampus. Acuna et al. also used this method to separate GABA, Glu and glutamine in rat brain homogenates with higher accuracy and repeatability (Monge-Acuña and Fornaguera-Trías, 2009). Reinhoud et al. (2013) used microbore columned Ultra-HPLC to detect catecholamines such as dopamine (DA) and serotonin (HT-5). The main benefit of this method was that analytes with large differences in retention time could be separated in a single run (Reinhoud et al., 2013). Commercial HPLC-ED systems (Alexys, Dionex) are now available that utilize GCEs at ∼0.8 V for amino acid detection. However, this state-of-the-art technology is bulky and unsuitable for real-time continuous GABA monitoring, which is a key technology gap in the chemical neuroscience field.

**FIGURE 1:**
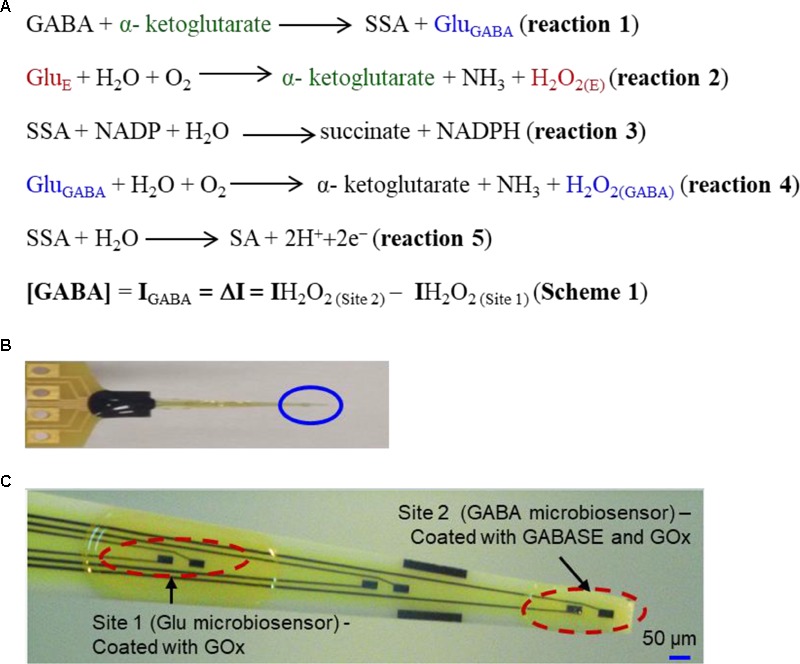
**(A)** Reaction pathways and detection scheme for real-time continuous GABA monitoring. **(B)** An optical image of the GABA probe. **(C)** The probe at higher magnification showing 6 of 8 Pt microelectrodes. Each site has two modified microelectrodes. For this work, Site 1 is modified with GOx (Glu microbiosensor) and Site 2 is modified with GOx and GABASE enzymes (GABA microbiosensor). Each Pt microelectrode is 100 μm × 50 μm. The distance between the sites is 1 mm. For recording in brain slices, the two Pt microelectrodes at the tip of the probe (marked Site 2) were modified with GOx and GABASE+ GOx, respectively (see Supplementary Material). The size of the microelectrode sites and distance between them was small enough to ensure that both microelectrodes were inserted completely within brain slices.

The second-best detection method is based upon an amperometry (AM) technique during which GABA is detected indirectly using biosensors (Mazzei et al., 1996; Badalyan et al., 2007, 2014; Sekioka et al., 2008). The main advantage of AM-based biosensors are that they can be easily miniaturized into multiple microarrays and are highly suitable for *ex vivo* and *in vivo* studies (Garguilo and Michael, 1994; Hascup et al., 2007). Biosensors selectively oxidize GABA into a secondary electroactive product or reporter molecule in the presence of enzymes, similar to the detection method used for Glu (Hascup et al., 2007) or acetylcholine (Garguilo and Michael, 1994). Electroactive reporter molecules such as β-nicotinamide adenine dinucleotide phosphate (NADPH) or hydrogen peroxide (H_2_O_2_) are usually generated through a series of enzymatic reactions by adding nicotinamide adenine dinucleotide phosphate (NADP) as a co-factor, or α-ketoglutarate reagents externally, and then electrochemically detecting them on a modified GCE. The current generated by electrochemically oxidizing them can be used as a quantifying index of GABA’s presence. In AM-based GABA biosensors, GABASE, which consists of two enzymes, γ-aminobutyrate aminotransferase (GABA-T) and succinic semialdehyde dehydrogenase (SSDH) converts GABA into Glu (henceforth called Glu_GABA_) and succinic semialdehyde (SSA) in the presence α-ketoglutarate (reaction 1, **Figure 1A**). For reaction 1 to occur, α-ketoglutarate must be present in the sample. The α-ketoglutarate can be added to the sample externally or it can be obtained from oxidizing Glu that is ubiquitously present in the brain microenvironment (henceforth called Glu_E_) using the glutamate oxidase (GOx) enzyme (reaction 2, **Figure 1A**). Subsequently, there are two pathways by which reaction 1 can proceed to electrochemically or optically generate active molecules that indicate the presence of GABA. The first approach is based on SSA reacting with NADP in the presence of SSDH to form NADPH that is then detected optically [UV spectrophotometry or colorimetry (Sethi, 1993) or electrochemically (reaction 3, **Figure 1A**)]. Mazzei et al. (1996) developed a GABA biosensor based on reaction 3 using a horseradish peroxidase-modified GCE. However, the main disadvantage of reaction 3 is that the electrode surface fouls rapidly due to the irreversible nature of NADP^+^ adsorption. Sekioka et al. (2008) partially addressed this challenge using an electron cyclotron resonance (ECR) sputtered carbon electrode. Since there is a critical need for *ex vivo* and *in vivo* studies to detect GABA long term and NADP to NADPH conversion is irreversible, NADP must be continuously replenished. Badalyan et al. (2007) addressed this problem of continual NADP additions by employing periplasmatic aldehyde oxidoreductase instead of SSDH, and mediators such as ferricyanide, phenoxazines, ferrocene derivatives, quinones, and bipyridinium salts instead of NADP.

**FIGURE 2:**
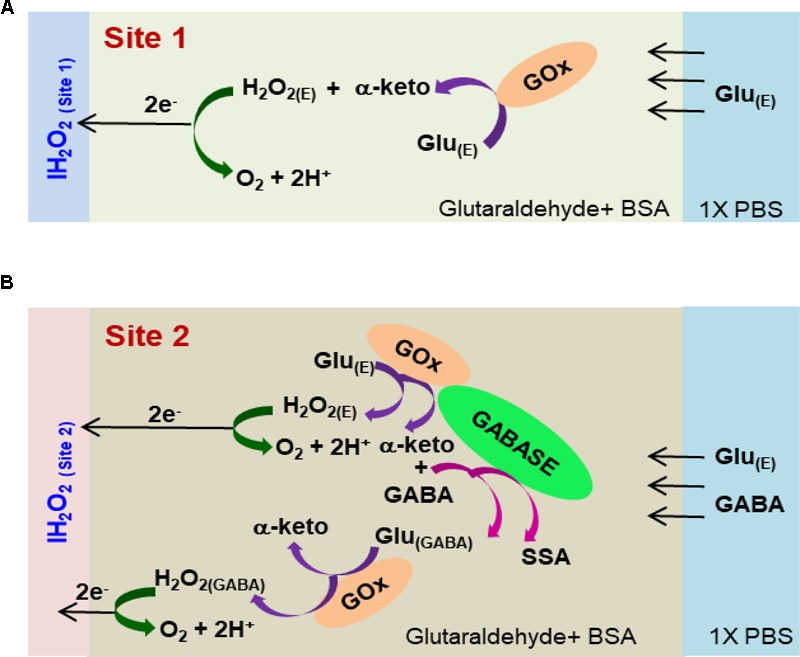
Schematic of the reaction pathways in **(A)** Glu microbiosensor (Site 1) and **(B)** GABA microbiosensor (Site 2).

Niwa et al. (1998) employed a very different approach that relied on GOx to convert the Glu_GABA_ generated in reaction 1 into α-ketoglutarate and H_2_O_2_ (henceforth called H_2_O_2(GABA)_, (i.e., H_2_O_2_ generated from the Glu that in turn is generated from GABA; reaction 4, **Figure 1A**) and then detecting it on an osmium-poly(vinylpyridine) gel-horseradish peroxidase-modified GCE. Applying reactions 1 and 4, researchers were able to detect GABA with adequate sensitivity and selectivity in the presence of DA, HT-5 and ascorbic acid (AA). However, both approaches are incapable of continuously monitoring the changes in GABA levels in real-time since they require additions of reagents such as NADP and α-ketoglutarate. A biosensor technology that can accurately measure GABA in real-time continuously and without any external intervention is technically challenging and yet unrealized. In this work, we report the development and validation of a GABA probe based upon a platinum (Pt) microelectrode array (MEA) (**Figure 1B**) in an *in vitro* setting and then used the probe for *ex vivo* measurements in brain slices. The GABA probe uses two types of microbiosensors, namely a Glu microbiosensor (located in Site 1, **Figure 1C**) and a GABA microbiosensor (located in Site 2, **Figure 1C**) that are uniquely modified with GOx only at Site 1 for reaction 2 to occur and with GOx and GABASE at Site 2 for reactions 1, 2, and **4** to occur. Each site in the GABA microarray probe consists of two Pt microelectrodes that are separated by 100 μm. By simultaneously measuring and subtracting the oxidation currents of H_2_O_2_ generated from the two microbiosensors, i.e., IH_2_O_2_ from H_2_O_2(E)_ at Site 1 (henceforth called IH_2_O_2(Site1)_) and IH_2_O_2_ from H_2_O_2(E)_ and H_2_O_2(GABA)_ at Site 2 (henceforth called IH_2_O_2(Site2)_), GABA (I_GABA_ = ΔI = IH_2_O_2(Site2)__-_ IH_2_O_2(Site1)_) can be detected continuously in real time (Scheme 1, **Figure 1A**) without adding α-ketoglutarate externally (**Figure 2**). This is possible because α-ketoglutarate generated in reaction 2 is used in reaction 1. Scheme 1 can be readily implemented *ex vivo* and *in vivo* because the ubiquitous presence of Glu_E_ allows *in situ* generation of α-ketoglutarate, and thus reaction 1 to occur continuously. The SSA generated in reaction 1 is converted to SA when periplasmatic aldehyde reductase is present on the electrode surface (reaction 5, **Figure 1A**) (Badalyan et al., 2014). The other salient features of the GABA probe are: (1) eight individually electrically addressable Pt microelectrodes that can easily be multiplexed to simultaneously measure other important neurochemicals, such as Glu, DA, adenosine and HT-5, through suitable surface modifications, which is not possible with other commonly available electrodes for chemical sensing, e.g., carbon fiber microelectrodes; (2) GABA and Glu microbiosensors can be placed in close proximity to provide precise measurements of local GABA level changes; (3) an ability to detect GABA real-time without adding reagents (i.e., truly self-contained); (4) the location of MEAs along the long shank allows GABA sensing at multiple depths in the brain; and (5) allows simultaneous sensing of neurochemicals and field potentials for multimodal recordings, which is not possible with the current neurochemical technologies.

## Materials and Methods

### Chemicals

Phosphate buffered saline (PBS), bovine serum albumin (BSA), glutaraldehyde, GABA, GABASE from Pseudomonas fluorescens and α-ketoglutarate disodium salt was purchased from Millipore-Sigma (MO, United States). Glutamate oxidase was purchased from Cosmo Bio United States (CA, United States).

### GABA Probe Preparation

The platinum (Pt) MEA (8-TRK probe) was purchased from Center for Microelectrode Technology (CenMeT, United States). The MEA consists of eight Pt microelectrodes (50 μm × 100 μm, two microelectrodes per site) and the sites are spaced at 1 mm apart. Each site has two closely spaced (100 μm apart) microelectrodes (**Figure 1C**). Since the *in vitro* experiments were carried out in a stirred solution in a beaker, we do not expect to see any effect or variability particularly on the Glu signal due to this spatial variation. For *ex vivo* measurements in brain tissue slices, the two Pt microelectrodes (located in Site 2, as shown in Supplementary Figure S3A, and spaced 100 μm apart, see Supplementary Material) were coated with GOx and GABASE+GOx, respectively.

#### Enzyme Aliquot Preparation

The GOx enzyme with the BSA and glutaraldehyde was coated in Site 1 as per Burmeister et al. (2013). For Site 1, the GOx enzyme was mixed in DI water to prepare aliquots of 0.5 U/μL and stored in -80°C. Prior to coating, they were thawed first at 4°C and then at room temperature. DI water (985 μL) was added to 10 mg BSA in a 1 mL centrifuge tube. After allowing the BSA to dissolve, 5 μL of glutaraldehyde (25% in water) was added to the solution. We kept the solution mixture (1% BSA and 0.125% glutaraldehyde) at room temperature for ∼5 min. A 4 μL of the mixture was added to 1 μL of GOx (0.5 U/μL) and centrifuged to form the final enzyme-matrix mixture of 0.1 U/μL GOx/0.8% BSA/0.1% glutaraldehyde. Similarly, for Site 2, DI water (986.7 μL) was added to 13.33 mg BSA in a 1 mL centrifuge tube. After allowing the BSA to dissolve, 6.67 μL of glutaraldehyde (25% in water) was added to the solution. We kept the solution mixture (1.33% BSA and 0.166% glutaraldehyde) at room temperature for ∼5 min. Next, 3 μL of the mixture was added to 1 μL of GOx (0.5 U/μL) and 1 μL GABASE (0.5 U/μL) and centrifuged to form the final enzyme-matrix mixture of 0.1 U/μL GOx/0.1 U/μL GABASE/0.8% BSA/0.1% glutaraldehyde. For the GABASE-only site, the procedure used for Site 1 was followed except that GABASE instead of GOx was used.

#### Enzyme Coating Procedure

Under a Nikon stereomicroscope (Model, SMZ18), three drops (0.05 μL/drop) of the respective enzyme-matrix mixture was applied manually at each site using a microsyringe (Hamilton^®^, Model 701 N). Then the probe was stored for 48 h in an aluminum foil covered storage container with no exposure to light prior to use. **Figure 2** shows the cross-sectional schematic of the GABA probe with reaction pathways in Sites 1 and 2.

### Electrochemical Measurements

For amperometry measurements, a multichannel FAST-16mkIII^®^ potentiostat (Quanteon, LLC, Nicholasville, KY, United States) in a 2-electrode setup was used with an Ag/AgCl electrode as the reference electrode. The applied potential was set at +0.7 V for H_2_O_2_ detection. Note: This applied potential can be reduced to +0.3 V vs. Ag/AgCl when modified using platinum black as reported in the literature (Ben-Amor et al., 2014). The experiment was carried out in a 40 mL buffer solution. The analytes were introduced into the solution using a syringe pump (KD Scientific, Legato^®^ 100 syringe pump) to obtain the desired concentrations (M). The solution was continuously stirred at 200 rpm and maintained at 37°C. All measurements were repeated 6 times (*n* = 6). The Fast analysis^®^ software provided by Quanteon was used for data analysis. Sensitivity was defined as the change in current for each unit of analyte addition. Sensitivity was calculated from the slope (pA/μM) of the calibration curves. Then the slope was converted into nAμM^-1^cm^-2^ by dividing it by the Pt microelectrode area (5 × 10^-5^ cm^2^). The limit of detection (LOD) was calculated by dividing (3 times the standard deviation of 10 points from the baseline) by the least squares slope, which is based on the FAST 2014 software manual provided by Quanteon. The baseline is the signal that was obtained when no electroactive analyte was present in the solution. Two-tailed Students *t*-test was performed (*n* = 6) at two different confidence intervals. They are 99.99% (*p* < 0.0001) and 95% (*p* < 0.05). The values lie within *p* < 0.0001 unless otherwise stated. The value which lies within *p* < 0.05 are represented with (^∗^) in the bar charts and tables. One-way ANOVA was performed (*n* = 6) with significance defined as *p* < 0.05 to verify if sensor-to sensor variation (in the same site) is significant. Error value is shown as mean ± SEM.

### Recording GABA and Glutamate in Brain Tissue

#### Animal Care and Use

Male Sprague Dawley rats were housed on a 12 h on – 12 h off cycle with food and water provided *ad libitum*, according to a Louisiana Tech University IACUC protocol, the Guide for the Care and Use of Laboratory Animals and the AVMA Guidelines on Euthanasia.

#### Hippocampal Slice Preparation

Hippocampal slices were prepared from an adult Sprague Dawley rat that was anesthetized using 5% isoflurane gas prior to decapitation and rapid removal of the brain. The brain was immediately placed into ice cold artificial cerebral spinal fluid (*a*CSF) containing (in mM): 135 NaCl, 3 KCl, 16 NaHCO_3_, 1 MgCl, 1.25 NaH_2_PO_4_, 2 CaCl_2_, and 10 glucose, bubbled with 95% O_2_/5% CO_2_ (carbogen) (Song et al., 2005). The slicing chamber of an OTS-5000 tissue slicer (Electron Microscopy Sciences) was filled with *a*CSF at 4° C and then 500-μm thick coronal sections were cut and transferred to a holding chamber filled with *a*CSF maintained at 35°C and bubbled with carbogen. Slices were incubated for at least 60 min prior to recording. Thereafter, one slice was transferred to a liquid-air interface of a BSC1 chamber (Scientific Systems Design, Inc.) with the slice suspended on a nylon net at the liquid-air interface with continuously dripping *a*CSF (37°C) bubbled with carbogen. Waste products were removed by continuous suction from the recording chamber (Supplementary Figure S3B, see Supplementary Material).

#### GABA Recording in Rat Hippocampal Slices

The microbiosensors were coated with a size-exclusion polymer (m-phenylenediamine, mPD) to prevent the interferents reaching the microbiosensor surface and to enhance the probe selectivity (Wilson et al., 2017). Supplementary Figure S4 (see Supplementary Material) demonstrates the ability of the mPD coating to block dopamine and ascorbic acid effectively. The tradeoff here is that, with an mPD coating, a ∼20% decrease in the sensitivity of the probe to GABA was observed (Supplementary Figure S5). Similar decrease in sensitivity values was observed at the Glu microbiosensor as well. The mPD layer was electrochemically deposited (cycling between +0.2 V and +0.8 V, 50 mV/s, 20 min in 10 mM mPD solution). A pair of 160-μm diameter tungsten stimulation electrodes was placed in the Schaffer collateral CA1 pathway within 200 μm of the microbiosensor probe sites (Song et al., 2005). An A365 stimulus isolator (World Precision Instruments) was used to deliver 100-μA direct current pulses to the stimulus electrodes; pulse widths were regulated by transistor-transistor logic (TTL) input from an Arduino microcontroller. Current detected at the probe sites was plotted in real time.

#### Data Analysis for *ex Vivo* Recordings

Results from *ex vivo*, hippocampal recordings were analyzed using OriginPro 2017. Measurements are reported as the mean ± square error of the mean (SEM). ANOVA was performed for comparisons of means and significance was defined as *p* ≤ 0.05. Rise times (T_r10-90_) were defined as the elapsed time between 10 and 90% from the baseline to the peak current of the stimulation response. The Rise Time Gadget tool in OriginPro 2017 was used to calculate the rise time.

## Results and Discussion

### Calibration of GABA Probe in the Presence of α-Ketoglutarate

**FIGURE 3:**
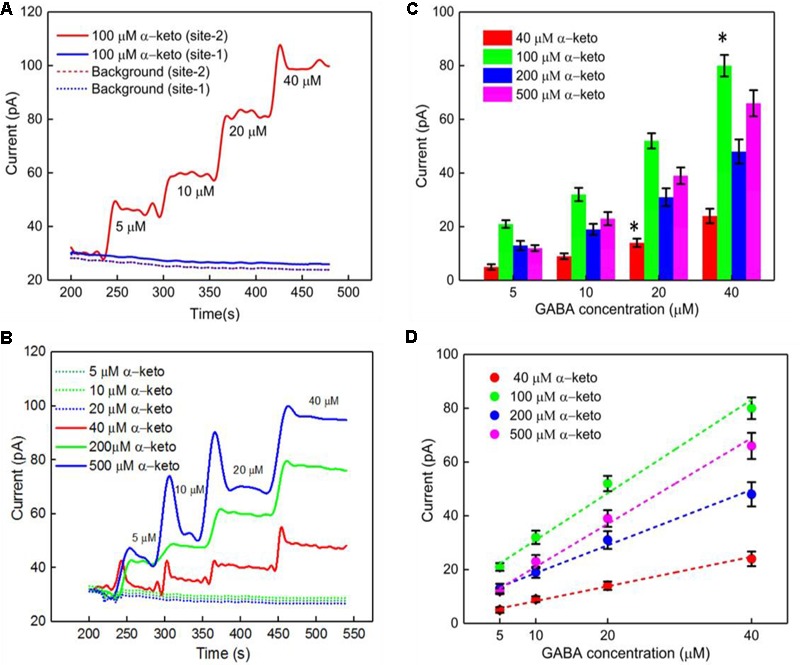
GABA probe calibration in different concentrations of GABA (5, 10, 20, and 40 μM) and α-ketoglutarate (5, 10, 20, 40, 100, 200, and 500 μM) in 1X PBS. **(A)** Current response at GABA microbiosensor in Site 2 and Glu microbiosensor in Site 1 in PBS only (background or control – red dashed curve, blue dashed curve, respectively) and in 100 μM α-ketoglutarate in 1X PBS (red and blue solid curves, respectively). **(B)** Current response at GABA microbiosensor for other concentrations of α-ketoglutarate. **(C,D)** Current response and linear fitting at GABA microbiosensor for different GABA concentrations and α-ketoglutarate concentrations at ≥ 40 μM. Legends: 40 μM (red), 100 μM (green), 200 μM (blue) and 500 μM (magenta). The microbiosensors were biased at + 0.7 V vs Ag/AgCl reference. The solution was stirred at 200 rpm and maintained at 37°C. Linear fit parameters obtained: 40 μM α-keto sensitivity = (0.55 ± 0.077 pA/μM), R^2^ = 0.99728; 100 μM α-keto sensitivity = (1.74 ± 0.13 pA/μM), R^2^ = 0.99582; 200 μM α-keto sensitivity = (1.03 ± 0.13 pA/μM), R^2^ = 0.99582 and 500 μM α-keto sensitivity = (1.51 ± 0.13 pA/μM); R^2^ = 0.99582 at GABA microbiosensor. Two-tailed Students *t*-test was performed (*n* = 6, *p* < 0.0001, ^∗^*p* < 0.05). One-way ANOVA was performed (*n* = 6, *p* < 0.0001) to verify that sensor-to sensor variation (in the same site) is not significant. Error value is shown as mean ± SEM.

Studies have shown dependence of the GABA current response (pA) on concentration of *α-*ketoglutarate (Niwa et al., 1998), which is an important molecule in physiological functions, for example in the Krebs cycle (Tretter and Adam-Vizi, 2005). Therefore, we first studied the electrochemical response of the Glu and GABA microbiosensors (Sites 1 and 2) in the presence of different concentrations of α-ketoglutarate (1–500 μM) in the phosphate buffered saline (PBS) solution. **Figure 3A** shows the typical AM responses at Sites 1 and 2 in 1X PBS supporting electrolyte (background or control, blue dashed, red dashed curves), and to varying concentrations of GABA (5, 10, 20 and 40 μM) in 100 μM α-ketoglutarate solution prepared in 1X PBS (blue solid, red solid curves). These values of concentration in the micromolar range were chosen because of their relevance to the ones encountered in the brain microenvironment where GABA is typically present (Badalyan et al., 2014). For example, GABA levels are in the range of 20–70 μM in rat brain slices, (Grabauskas, 2004), and up to 1.25 μM/cm^3^ in the human brain (Ke et al., 2000) as measured by proton magnetic resonance spectroscopy. The AM response was recorded in different concentrations of α-ketoglutarate solution, first by allowing the microbiosensors to stabilize in the solution for up to 240 s, and then injecting GABA at 1 min time intervals to obtain the desirable concentration (**Figures 3B,C**). From **Figure 3A**, as expected, we observe that the Glu microbiosensor at Site 1 did not exhibit a response to GABA because of the absence of the GABASE enzyme. Also, there was no enzymatic activity of GOx in converting GABA into Glu and then into H_2_O_2_. This indicates that the GABA conversion is highly selective at Site 2 that has GABASE and not at Site 1. The GABA microbiosensor at Site 2 responded to GABA when the α-ketoglutarate concentration was at least 40 μM (**Figure 3B**). A transient spike in the signal was observed during the injection of the solution in the beaker. However, the signal was stabilized a few seconds following the injection of the solution. Sometimes the time to stabilization was a bit longer (e.g., in the case of 40 μM and 500 μM α-ketoglutarate experiments). This might be due to a few bubbles in the micro syringe pump that disturb the solution more in certain experiments than others. The other data points for the same α-ketoglutarate concentrations did not show similar spikes. The highest sensitivity was observed at 100 μM. From **Figure 3D**, the sensitivity is 36 ± 2.5 pA μM^-1^ cm^-2^ and the LOD is 2 ± 0.12 μM (*n* = 6), which is 10-fold higher than that of similar AM-based microsensors (Niwa et al., 1998). The sensitivities at 40, 200, and 500 μM of α-ketoglutarate were 12 ± 1.7, 20 ± 2.4 and 28 ± 2.5, respectively, and the LOD was 7 ± 0.7, 4.0 ± 0.4, and 3 ± 0.24, respectively (**Table 1**). This GABA response to α-ketoglutarate concentration is in agreement with previously published literature (Niwa et al., 1998). One possible reason for the decrease of GABA sensitivity at highest α-ketoglutarate concentrations could be due to their scavenging of H_2_O_2_ as suggested by previous studies (Nath et al., 1995; Long and Halliwell, 2011). Another study (Badalyan et al., 2014) showed a similar trend where the GABA sensitivity was highest at 1 mM α-ketoglutarate and then decreased at much higher concentrations. The LOD achieved using the GABA microbiosensor is 2–7 μM, which is lower than the clinically-relevant concentrations (Grabauskas, 2004) and similar to the values achieved by alternative methods (Ke et al., 2000) in the human brain. Sensitivities differ slightly between microelectrodes, which are likely due to variations in the quantity of enzymes that are manually applied to each site. Any potential defects in the surface of the electrodes may also lead to a difference in sensitivity. But this could be remedied by employing an array of GABA and Glu microbiosensors and by applying appropriate statistics (e.g., averaging the current values, etc.) in the future. This sensitivity variation can be further minimized by employing micro spotting techniques that are fully automated and dispense very precise volumes of enzyme solutions. Next, to determine the linear range of the calibration plots, we generated the plots for 5–500 μM GABA concentrations versus different α-ketoglutarate concentrations. We observe that the GABA current values saturate, and saturation depends on the α-ketoglutarate concentration (Supplementary Figures S1, S2, see Supplementary Material). For example, for 40 μM α-ketoglutarate, the GABA signal saturation is at 50 μM. Whereas in 100, 200 and 500 μM α-ketoglutarate concentrations, the GABA signal saturation occurs at 100 μM. The trend in sensitivity in the linear range is the same as before. For 100 μM α-ketoglutarate, the GABA sensitivity is highest and becomes lower at other concentrations of α-ketoglutarate.

**Table 1:** GABA sensitivity and LOD for different α-ketoglutarate concentration.

α-ketoglutarate concentration (μM)	Sensitivity (nA μM^-1^cm^-2^)	LOD (μM)
40	12 ± 1.7	7 ± 0.7
100	36 ± 2.5	2 ± 0.12
200	20 ± 2.4^∗^	4 ± 0.4
500	28 ± 2.5	3 ± 0.24^∗^

*Error value is shown as mean ± SEM. Two-tailed Students *t*-test was performed (*n* = 6, *p* < 0.0001, ^∗^*p* < 0.05). One-way ANOVA was performed (*n* = 6, *p* < 0.0001) to verify that sensor-to sensor variation (in the same site) is not significant.*

### Calibration of the GABA Probe in the Presence of Glutamate

**FIGURE 4:**
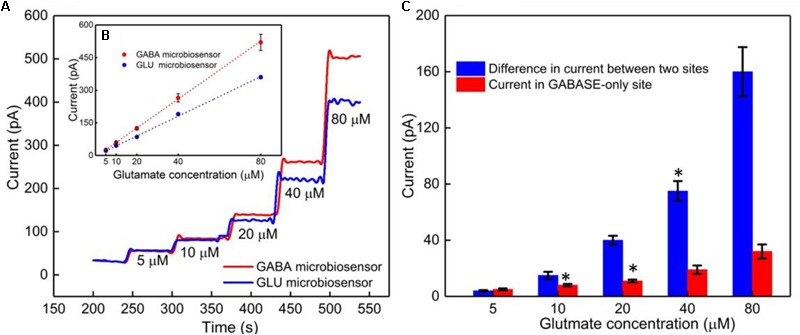
GABA probe calibration in different concentrations of Glu (5, 10, 20, 40, and 80 μM). **(A)** Current response at GABA microbiosensor in Site 2 and Glu microbiosensor in Site 1 (red and blue solid curves, respectively). **(B)** Inset showing the linear fitting for GABA and Glu microbiosensors (red and blue dotted lines). Linear fit parameters obtained: Site 2, GABA microbiosensor: Glutamate sensitivity = (6.67 ± 0.38 pA/μM), R^2^ = 0.99984; Site 1, Glu microbiosensor: Glutamate sensitivity = (4.55 ± 0.11 pA/μM), R^2^ = 0.99926. **(C)** The difference in the current response between the microbiosensors (blue bars). The current response at GABA microbiosensor that was coated with GABASE enzyme only (no GOx enzyme) (red bars). Two-tailed Students *t*-test was performed (*n* = 6, *p* < 0.0001, ^∗^*p* < 0.05). One-way ANOVA was performed (*n* = 6, *p* < 0.0001) to verify that sensor-to sensor variation (in the same site) is not significant. Error values are shown as mean ± SEM. Note: the error bars are too small for the blue dotted data for the naked eye to see. The microbiosensors were biased at + 0.7 V with respect to an Ag/AgCl reference electrode. The solution was stirred at 200 rpm and maintained at 37°C. No α-ketoglutarate was added during any of the experiments.

The GABA probe was calibrated in the presence of a range of concentrations (5–80 μM) of Glu, which mimics the brain microenvironment both in healthy and diseased states. For example, the basal concentration of Glu in the extracellular space is up to 20 μM (Moussawi et al., 2011), while Glu concentrations in cerebrospinal fluid are ∼10 μM. During seizures, Glu levels increase 4-fold and GABA levels decrease (Rowley et al., 1995a; Kanamori and Ross, 2011; Medina-Ceja et al., 2015). Glu is a major excitatory neurochemical that is ubiquitously present as L-glutamate in its anionic form (glutamic acid) in the brain environment (henceforth called Glu_E_) (Moussawi et al., 2011). One of the objectives of this study was to monitor Glu_E_ as an *in-situ* source for the generation of α-ketoglutarate, which aids in the continuous real-time GABA monitoring at Site 2, and thus does not rely on the addition of α-ketoglutarate externally. Firstly, we calibrated the two microbiosensors by injecting Glu at various concentrations (5, 10, 20, 40, and 80 μM) in 1X PBS buffer solution. **Figures 4A,B** shows the response of the two microbiosensors. The GABA microbiosensor (Site 2) consistently exhibited a slightly higher Glu response than that of the Glu microbiosensor (Site 1). The Glu sensitivity of Site 2 and Site 1 are 132 nA μM^-1^cm^-2^ and 90 nAμM^-1^cm^-2^, respectively. The difference in the current response from the two microbiosensors increases for higher Glu concentrations (**Figure 4C**, blue bars). To further understand this, we modified Site 2 with only GABASE and no GOx. Ideally, there should not be any response from the GABA microbiosensor, however, a small response was observed (**Figure 4C**, red bars). This confirms our hypothesis that some non-selective activity of GABASE is due to Glu oxidation. Others have made similar observations where GABASE showed weak enzyme activity toward Glu compared to GOx (Niwa et al., 1998). The large response could also be due to the presence of more enzymes per unit volume (0.2 U/μl) that somehow collectively create more active sites (Arima et al., 2009). To account for this difference in the Glu response, henceforth called the background noise, I_b_ [shown in **Figure 4C** (blue bars)], the I_b_ was subtracted from the difference in the currents (I_GABA_) at the two sites in order to obtain the final current response to GABA (details discussed later).

**FIGURE 5:**
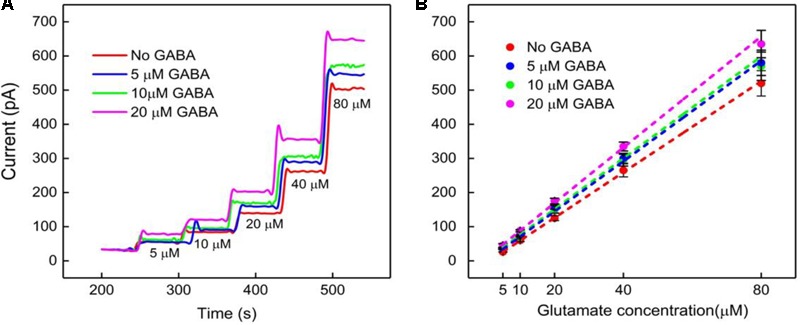
GABA probe calibration in different concentrations of Glu (5, 10, 20, 40, and 80 μM). **(A,B)** Current response and linear fitting at GABA microbiosensor in Site 2, with and without GABA. Legends: no GABA (red solid curve), 5 μM GABA (blue), 10 μM GABA (green) and 20 μM GABA (magenta). Linear fit parameters obtained for Glu with different concentration of GABA: no GABA (red line), Glu sensitivity = (6.6 ± 0.06 pA/μM), R^2^ = 0.99973; 5 μM GABA (blue) Glu sensitivity = (7.3 ± 0.11 pA/μM), R^2^ = 0.99921; 10 μM GABA (green), Glu sensitivity = (7.7 ± 0.12 pA/μM); R^2^ = 0.99927 and 20 μM GABA (magenta), GLU sensitivity = (8.2 ± 0.17 pA/μM); R^2^ = 0.99863 at GABA microbiosensor. Two-tailed Students *t*-test was performed (*n* = 6, *p* < 0.0001). One-way ANOVA was performed (*n* = 6, *p* < 0.0001) to verify that sensor-to sensor variation (in the same site) is not significant. Error value is shown as mean ± SEM. The microbiosensor was biased at + 0.7 V vs Ag/AgCl reference. The solution was stirred at 200 rpm and maintained at 37°C. No α-ketoglutarate added during all the experiments.

The next calibration step was to test different GABA solutions (0, 5, 10, and 20 μM) in 1X PBS buffer and repeat the above Glu calibration (**Figure 5**). These experiments were performed without adding α-ketoglutarate externally. At Site 1, Glu_E_ is oxidized to α-ketoglutarate and H_2_O_2(E)_ (reaction 2). This α-ketoglutarate then reacts with GABA at Site 2 and produces Glu_GABA_ (reaction 1) followed by reaction 4, which generates H_2_O_2(GABA)_ and more α-ketoglutarate. These reactions and pathways were shown in **Figure 2**. At the GABA microbiosensor (Site 2), in the case of no GABA in the solution, the current response (IH_2_O_2(Site2)_) is due only to the changing Glu levels in the solution (**Figure 5A**, red curve). When GABA is present in the solution, the IH_2_O_2(Site2)_ response is from both GABA and Glu oxidation and we expect it to be larger than the response when there was no GABA. Therefore, higher GABA concentrations appear to induce a greater response (**Figure 5A**, blue, green, and magenta curves) at Site 2 and greater I_GABA_, which is the GABA signal (Scheme 1). **Figure 5B** shows the sensitivity of the GABA microbiosensor at different GABA and Glu concentrations. With increasing GABA and Glu concentrations, the sensitivity of the GABA microbiosensor increases and this is because of increased availability of α-ketoglutarate for reaction 1. The sensitivity and the LOD of the two microbiosensors is shown in **Table 2**. The GABA sensitivity increased by ∼25% at 20 μM GABA concentrations. The sensitivity reported here is greater than that of the Pt based Glu sensors published in the literature (Tseng et al., 2014) The LOD is comparable to other Glu sensors (Khan et al., 2011).

**Table 2:** Sensitivity and LOD in Site 1 (GOx only) and Site 2 (GOx + GABAse).

GABA concentration (μM)	Sensitivity (nA μM^-1^cm^-2^)		LOD (μM)	
	
	Site-1	Site-2	Site-1	Site-2
0	90 ± 5^∗^	132 ± 13	0.27 ± 0.02	0.08 ± 0.008
5	104 ± 8	146 ± 16	0.23 ± 0.01	0.08 ± 0.007^∗^
10	106 ± 10	154 ± 19	0.22 ± 0.01	0.07 ± 0.008
20	104 ± 12	164 ± 21	0.23 ± 0.02	0.07 ± 0.009

*Error value is shown as mean ± SEM. Two-tailed Students *t*-test was performed (*n* = 6, *p* < 0.0001, ^∗^*p* < 0.05). One-way ANOVA was performed (*n* = 6, *p* < 0.0001) to verify that sensor-to sensor variation (in the same site) is not significant.*

### Quantification of GABA Using the I_GABA_ and IH_2_O_2(E)_ Current Values

**FIGURE 6:**
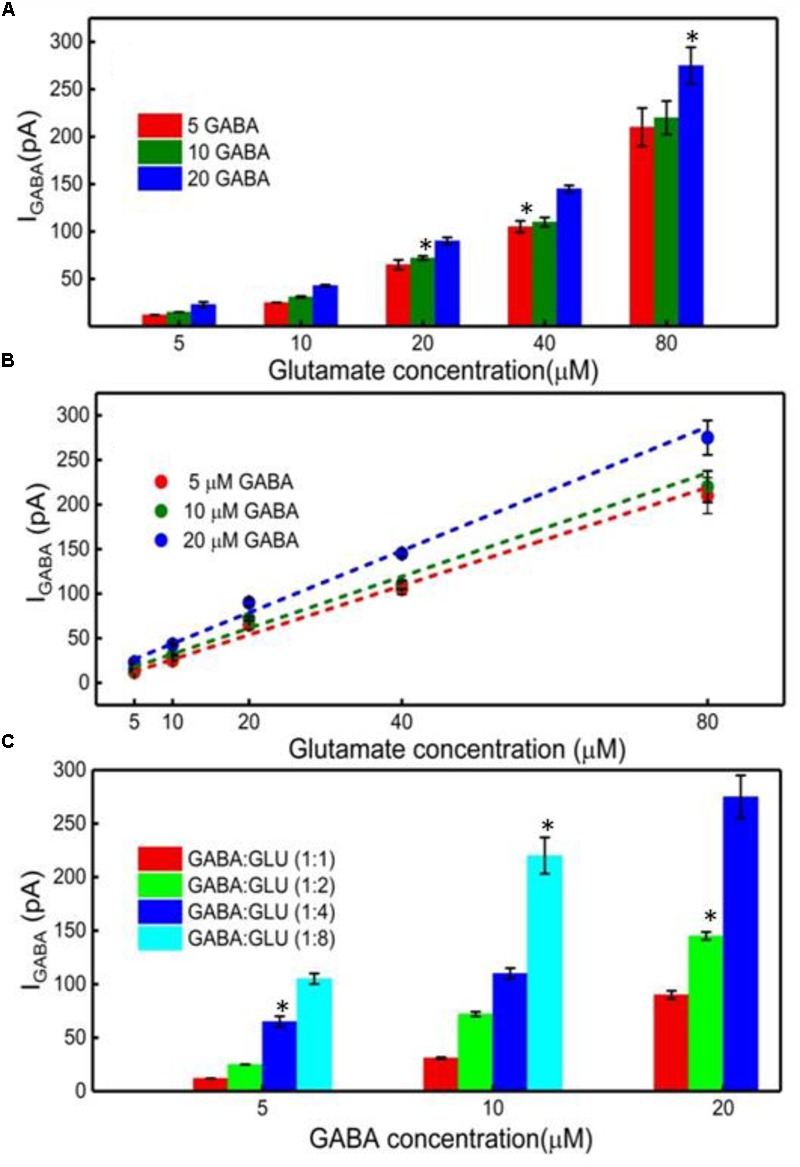
GABA detection using the GABA and Glu microbiosensors. **(A)** The I_GABA_ values for different GABA (5, 10, and 20 μM) and Glu concentrations (5, 10, 20, 40, and 80 μM). This value is taken after subtracting the baseline value, which is the difference in the current response between the microbiosensors without GABA in the solution. Legends: 5 μM GABA (red bar), 10 μM (green bar) and 20 μM (blue bar). **(B)** Linear fitting of I_GABA_. Linear fit parameters obtained: 5 μM GABA: slope = 2.75 ± 0.20 pA/μM, R^2^ = 0.99730; 10 μM GABA: slope = 2.90 ± 0.28 pA/μM, R^2^ = 0.99653; 20 μM GABA: slope = 3.47 ± 0.22 pA/μM, R^2^ = 0.99784. **(C)** The I_GABA_ values at different GABA:Glu molarity ratios. Legends: GABA:Glu = 1:1 (red), 1:2 (green), 1:4 (blue) and 1:8 (cyan bar). Two-tailed Students *t*-test was performed (*n* = 6, *p* < 0.0001, ^∗^*p* < 0.05). One-way ANOVA was performed (*n* = 6, *p* < 0.0001) to verify that sensor-to sensor variation (in the same site) is not significant. Error value is shown as mean ± SEM.

Finally, the GABA signal was quantified as I_GABA_ = IH_2_O_2(Site1)__-_ IH_2_O_2(Site2)_. The I_GABA_ is plotted for varying GABA and Glu concentrations in **Figure 6A** after subtracting the I_b_ noise. The positive values for I_GABA_ at all concentrations of GABA and Glu confirms GABA detection at Site 2. As expected, the I_GABA_ increases as GABA concentrations increase. The GABA calibration curves, following linear approximation of I_GABA_ at various Glu concentrations, is shown in **Figure 6B**. A steeper slope is evident at higher GABA concentrations. Values of the slope are 2.7 ± 0.2 pA/μM, 2.9 ± 0.3 pA/μM and 3.5 ± 0.2 pA/μM for 5, 10, and 20 μM GABA, respectively. To better understand the GABA signal dependence on Glu concentrations, I_GABA_ values were plotted in terms of different molarity ratios of GABA:Glu (1:1, 1:2, 1:4, and 1:8) for different GABA concentrations (**Figure 6C**). It is known that GABA and Glu maintains a certain balance in the human brain by means of the glutamate-glutamine (GABA) cycle (Hertz, 2013) And they exist in a certain molarity ratio based upon the state of the brain. For example, in epilepsy, this cycle becomes imbalanced and Glu levels are elevated (Rowley et al., 1995a; Kanamori and Ross, 2011; Medina-Ceja et al., 2015). The data clearly suggest that the I_GABA_ value is greatly dependent on both GABA and Glu concentration, i.e., the I_GABA_ increases as GABA and Glu levels increases. This is evident from **Figure 6C**, which shows that, for a given GABA concentration, the I_GABA_ value is larger for higher GABA:Glu ratios. So, in this approach, for a given I_GABA_ value, the GABA concentration can vary. For example, for an I_GABA_ value of 100 pA, the GABA concentration can be 5, 10, or 20 μM. This is because the GABA signal is dependent on the local availability of α-ketoglutarate, which is dependent on the local Glu concentration. Thus, there is no one I_GABA_ value for a given GABA concentration. This problem can be solved by considering the I_GABA_ value from Site 2 and the IH_2_O_2(E)_ value from Site 1. From the IH_2_O_2(E)_ value, the local Glu concentration can be measured. Once the local Glu concentration is known, (x-coordinate in **Figure 6B**), and since the I_GABA_ value is already known (y-coordinate in **Figure 6B**), their intersection yields the local GABA concentration. For example, let us say that the IH_2_O_2(E)_ value from the Glu microbiosensor is 175 pA and then from **Figure 4B**, the Glu concentration will be 50 μM. And, this 50 μM Glu is the x-coordinate in **Figure 6B**. Next, let us say that the I_GABA_ value is 175 pA, which is the difference between the IH_2_O_2_ values obtained from the two microbiosensors. Again, the I_GABA_ value is the y-coordinate in **Figure 6B**. So, from **Figure 6B**, with (x, y) as (50 μM, 175 pA), the intersection of the lines falls on the blue dashed line that corresponds to a GABA concentration of 20 μM.

Finally, in this work, for the *in vitro* experiments, the microbiosensors were not coated with selective coatings such as nafion and m-phenylenediamine (mPD) that have shown to completely block potential electroactive interferents such as dopamine and ascorbic acid. For the *ex vivo* testing, we coated the microbiosensors with mPD to achieve selectivity of the probe (Wilson et al., 2017).

### Real-Time Measurement of GABA and Glutamate in Rat Hippocampal Slice Preparation

**FIGURE 7:**
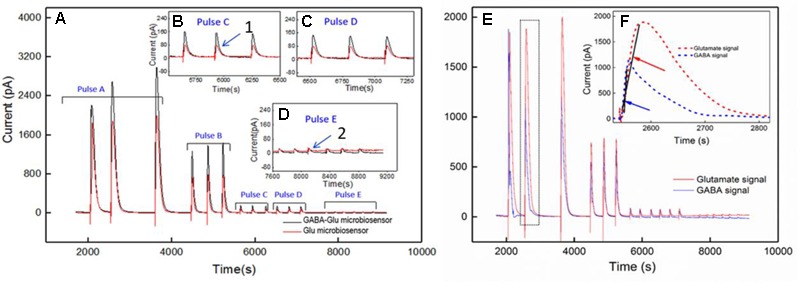
*Ex vivo* recording of stimulated release of Glu and GABA in rat hippocampal slice preparation. The amperometry method was used to record current with the microbiosensor biased at + 0.7 V with respect to an Ag/AgCl reference electrode. **(A)** Current responses to unipolar stimulation (tungsten wires, 100 μA) were recorded on a F.A.S.T. 16mkIII system (Quanteon, Kentucky; red traces, Glu microbiosensor; black traces, GABA microbiosensor). Stimulation pulse parameters (Pulse A–E) are listed in **Table 3** and range from 1 s to 5 ms. Conversion of peak current measurements to Glu and GABA concentrations are listed in **Table 4** for points 1 and 2 (see numbers with arrows). Insets **B, C,** and **D** show details of the responses to shorter pulse widths. **(E)** Processed GABA signal with Glu signal from responses to Pulse **C–E** stimulations in 7A. The GABA trace (blue trace) is the difference between the signals from the GABA-glutamate and the Glu microbiosensor sites (red trace). **(F)** Inset shows the rise time, t_r10-90_, for the GABA signal (blue curve) and the Glu signal (red curve) from the boxed region in E. Arrows indicate the slope of line drawn from t_r10-90_ for GABA (blue arrow points to line) and t_r10-90_ for Glu (red arrow points to line). The rise times for GABA were faster than for Glu, as the difference in the slopes of the lines illustrate.

Simultaneous and continuous real-time detection of GABA and glutamate was accomplished using electrically stimulated release in a hippocampal slice model. We used a range of 100-μA pulse widths to induce release of the neurotransmitters (see **Table 3**) to determine the responsiveness of the sensor to varying levels of stimulation which included single pulses ranging from 1 s to 25-ms in duration and a pulse train of ten 5-ms pulses. The GABA signal was derived by subtracting the signal from the Glu microbiosensor from the GABA microbiosensor. As expected, the amplitude of GABA and glutamate release scaled with pulse width (**Figure 7**). In some cases, GABA had a shorter peak duration, and in all cases the concentration of GABA rose faster than glutamate concentration (**Figure 7F**). For example, the mean rise time (± SEM) for a 25-ms stimulation was 3.12 ± 0.35 s for GABA and 6.94 ± 0.9 s for glutamate (*n* = 6, *p* < 0.05). Both GABA and glutamate leak out of neuronal synapses after neurons release these neurotransmitters. Mechanisms exist to quickly scavenge and recycle these neurotransmitters, but some molecules diffuse through the extracellular space (Danbolt, 2001; Robinson and Jackson, 2016; Boddum et al., 2016). Thus, there is a slight delay from stimulation to response, as well as a long decay period as GABA and glutamate are eventually cleared. Both of these dynamic processes are evident in the traces shown in **Figure 7** with a rapid, but not immediate increase in neurotransmitter concentration, and a slower decline to baseline representing release and uptake, respectively.

**Table 3:** Stimulation pulse parameters and rise time of the stimulated response.

Pulse ID	Pulse parameters	Glutamate t_r10-90_ (s)	Glutamate – GABA t_r10-90_ (s)	GABA Δt_r10-90_ (s)
A	1000 ms single pulse	25 ± 2.2	17 ± 1.24	8 ± 1.2
B	250 ms single pulse	19 ± 1.9	14 ± 1.1	5 ± 0.45
C	50 ms single pulse	12 ± 1	7 ± 0.85	5 ± 0.6
D	Ten 5-ms pulses separated by 1 ms	12 ± 1	7 ± 0.8	5 ± 0.75
E	25 ms single pulse	7 ± 0.9^∗^	4 ± 0.25^∗^	3 ± 0.35^∗^

*Values are expressed in mean ± SEM. Two-tailed *t*-test was performed (*n* = 3, *p* < 0.05). ^∗^Two-tailed *t*-test was performed (*n* = 6, *p* < 0.05).*

**FIGURE 8:**
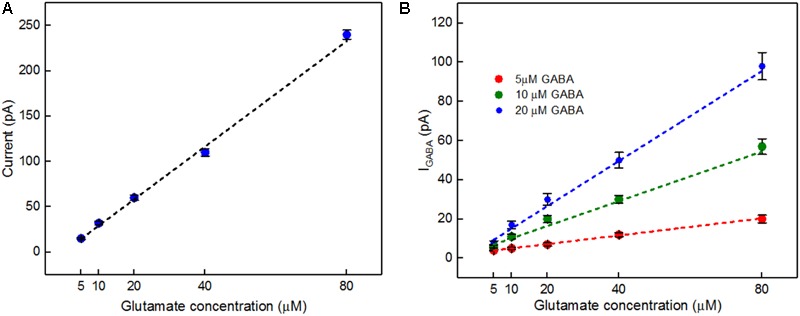
*In vitro* current signals for Glu and I_GABA_ taken from the GABA probe before the *ex vivo* recordings. **(A)** Current signals for varying Glu concentrations (5, 10, 20, 40, and 80 μM, blue dots) and the linear fit (black dashed). Linear fit parameters: Glu sensitivity: 2.91 ± 0.15 pA/μM, R^2^= 0.99364. **(B)** I_GABA_ for varying Glu concentrations. Legend: 5 μM GABA (red dots), 10 μM GABA (green dots) and 20 μM GABA (blue dots); linear fit for 5 μM GABA (red dashed), linear fit parameters: slope = 0.21 ± 0.006 pA/μM, R^2^= 0.99699, the linear fit for 10 μM GABA (green dashed), linear fit parameters: slope = 0.63 ± 0.09 pA/μM, R^2^= 0.9699 and linear fit for 20 μM GABA (blue dashed), linear fit parameters: slope = 1.14 ± 0.2 pA/μM, R^2^= 0.98967. Values are expressed in mean ± SEM. Two-tailed *t*-test was performed (*n* = 3, *p* < 0.05).

A calibration curve was constructed before performing the *ex vivo* recordings in order to convert current from GABA release to GABA concentration at the probe (**Figure 8A**). This calibration curve is constructed based on the procedure detailed in **Figure 4B**. The data plotted in **Figure 8B** is constructed in the same way as that of **Figure 6B**. Peak current measurements in **Table 4** represent a range of stimulated release of GABA and glutamate. These measurements correspond to curves labeled 1–2 in **Figure 7A**. Peak concentrations ranged from 5 to 35 μM for glutamate and 5–13 μM for GABA. Thus, these probes can measure GABA and glutamate at concentrations that are well below normal levels (Grabauskas, 2004; Moussawi et al., 2011) making them suitable to study impaired release in disease states. Furthermore, numerous cycles of stimulated release with consistent current amplitude for each level of stimulation and without adding any exogenous substrates, such as a-ketoglutarate, support the premise that endogenous products of the conversion of glutamate provide the substrate for the GABASE reaction. This is an important capability for future *in vivo* applications.

**Table 4:** Conversion of current to glutamate and GABA concentration in *ex vivo* recordings.

Points^1^	Glutamate Signal (pA)^2^	Glutamate (μM)^3^	Difference in signal (pA)^4^	GABA (μM)^5^
1	74	35	25	13
2	10	5	6	5

*^*1*^Number corresponds to signal trace number in **Figure 7A**. ^*2*^From **Figure 7E**, the IH_*2*_O_*2*_(E) value, i.e., the local Glu signal is measured. ^*3*^Then the local Glu concentration is known from **Figure 8A**. ^*4*^The I_GABA_ value is the difference between the IH_*2*_O_*2*_ values obtained from the two microbiosensors. ^*5*^Now, knowing the Glu concentration, which is the x-coordinate in **Figure 8B** and the I_GABA_ value, which is the y-coordinate in **Figure 8B**, one can find the GABA concentration for the two points.*

## Conclusion

In this work, we report a novel GABA microarray probe that can detect GABA without the addition of any external reagents such as α-ketoglutarate and NADPH *in vitro*. The GABA probe consists of two microbiosensors that were modified with GOx and GOx+GABASE enzymes. By simultaneously measuring and subtracting the oxidation currents of H_2_O_2_ generated from the microbiosensors. GABA was detected with a sensitivity of 36 ± 2.5 pA μM^-1^cm^-2^ and LOD of 2 ± 0.12 μM. We demonstrate a new detection method that will assist neuroscientists to better understand the combined role of GABA (a major inhibitory neurochemical) and Glu (a major excitatory neurochemical) in real-time in the brain. The key benefits of the proposed approach are: (1) the probe can be easily multiplexed to simultaneously measure other important neurochemicals, which is not possible with other commonly used electrodes for chemical sensing, (2) ability to detect GABA in real time without adding reagents (i.e., truly self-contained), (3) it is based on an established, commercially available Pt MEA platform that is suitable for future *in vivo* recordings, (4) the location of the MEAs along the long shank allows GABA and Glu sensing at multiple depths in the brain, and (5) it can simultaneously sense neurochemicals and field potentials for multimodal (e.g., neurochemical and neuroelectrical) recordings, which is not possible with the current neurochemical technologies. Furthermore, we demonstrated the utility of the microbiosensor microarray to simultaneously record fluctuations in electrically stimulated GABA and glutamate release continually and in real time in a rat hippocampal slice preparation. Moreover, we have shown that GABA release can be detected over repeated stimulations without adding substrate compounds externally. Future work for testing the GABA probe using *in vivo* animal models is anticipated.

## Author Contributions

IH designed the experiments, performed the experiments, analyzed the data, prepared the figures and manuscript. CT performed the experiments. PD performed the *ex vivo* experiments and analyzed the data. GD analyzed the *in vitro* data and figures. TM designed the *ex vivo* experiments, analyzed the data and prepared the *ex vivo* section of the manuscript. SS designed the *in vitro* experiments, analyzed the data and reviewed the manuscript. LI provided guidance and reviewed the manuscript. PA conceptualized and designed the GABA probe, detection scheme and experiments, provided guidance, prepared the figures, analyzed the data, and prepared manuscript.

## Conflict of Interest Statement

A report of invention (ROI 2017-09) is filed with Louisiana Tech University’s Office of Intellectual Property and Commercialization and filed a provisional patent. The authors declare that the research was conducted in the absence of any commercial or financial relationships that could be construed as a potential conflict of interest.

## References

[B1] Arima J. Sasaki C. Sakaguchi C. Mizuno H. Tamura T. Kashima A. (2009). Structural characterization of L-glutamate oxidase from *Streptomyces* sp. X-119-6. *FEBS J.* 276 3894–3903. 10.1111/j.1742-4658.2009.07103.x 19531050

[B2] Auteri M. Zizzo M. G. Serio R. (2015). The GABAergic system and the gastrointestinal physiopathology. *Curr. Pharm. Des.* 21 4996–5016. 10.2174/1381612821666150914121518 26365138

[B3] Badalyan A. Dierich M. Stiba K. Schwuchow V. Leimkühler S. Wollenberger U. (2014). Electrical wiring of the aldehyde oxidoreductase PaoABC with a polymer containing osmium redox centers: biosensors for benzaldehyde and GABA. *Biosensors* 4 403–421. 10.3390/bios4040403 25587431 PMC4287710

[B4] Badalyan A. Leimkühler S. Stiba K. Wollenberger U. (2007). *Biosensor for Measuring GABA. EP2505658A1.* 10.3390/bios4040403 PMC4287710 25587431

[B5] Ben-Amor S. Vanhove E. Belaïdi F. S. Charlot S. Arbault S. (2014). Enhanced detection of hydrogen peroxide with platinized microelectrode arrays for analyses of mitochondria activities. *Electrochim. Acta* 126 171–178.

[B6] Bhat R. Axtell R. Mitra A. Miranda M. Lock C. Tsien R. W. (2010). Inhibitory role for GABA in autoimmune inflammation. *Proc. Natl. Acad. Sci. U.S.A.* 107 2580–2585. 10.1073/pnas.0915139107 20133656 PMC2823917

[B7] Boddum K. Jensen T. P. Magloire V. Kristiansen U. Rusakov D. A. Pavlov I. (2016). Astrocytic GABA transporter activity modulates excitatory neurotransmission. *Nat. Commun.* 25:13572. 10.1038/ncomms13572 27886179 PMC5133667

[B8] Burmeister J. J. Davis V. A. Quintero J. E. Pomerleau F. Huettl P. Gerhardt G. A. (2013). Glutaraldehyde cross-linked glutamate oxidase coated microelectrode arrays: selectivity and resting levels of glutamate in the CNS. *ACS Chem. Neurosci.* 4 721–728. 10.1021/cn4000555 23650904 PMC3656760

[B9] Cahill P. S. David Walker Q. Finnegan J. M. Mickelson G. E. Travis E. R. Wightman R. M. (1996). Microelectrodes for the measurement of catecholamines in biological systems. *Anal. Chem.* 68 3180–3186. 10.1021/AC960347D 8797378

[B10] Caudill W. L. Houck G. P. Wightman R. M. (1982). Determination of gamma-aminobutyric acid by liquid chromatography with electrochemical detection. *J. Chromatogr.* 227 331–339. 10.1016/S0378-4347(00)80387-4 7061649

[B11] Cifuentes Castro V. H. López Valenzuela C. L. Salazar Sánchez J. C. Peña K. P. López Pérez S. J. Ibarra J. O. (2014). An update of the classical and novel methods used for measuring fast neurotransmitters during normal and brain altered function. *Curr. Neuropharmacol.* 12 490–508. 10.2174/1570159X13666141223223657 25977677 PMC4428024

[B12] Danbolt N. C. (2001). Glutamate uptake. *Prog. Neurobiol.* 65 1–105. 10.1016/S0301-0082(00)00067-8 11369436

[B13] Garguilo M. G. Michael A. C. (1994). Quantitation of choline in the extracellular fluid of brain tissue with amperometric microsensors. *Anal. Chem.* 66 2621–2629. 10.1021/ac00089a006 7943733

[B14] Grabauskas G. (2004). Time course of GABA in the synaptic clefts of inhibitory synapses in the rostral nucleus of the solitary tract. *Neurosci. Lett.* 373 10–15. 10.1016/j.neulet.2004.09.051 15555768

[B15] Hascup K. N. Rutherford E. C. Quintero J. E. Day B. K. Nickell J. R. Pomerleau F. (2007). “Second-by-second measures of L-glutamate and other neurotransmitters using enzyme-based microelectrode arrays,” in *Electrochemical Methods for Neuroscience* eds Micheal A. C. Borland L. M. (Boca Raton, FL: CRC Press/Taylor & Francis) 407–450. 21204381

[B16] Hertz L. (2013). The glutamate–glutamine (GABA) Cycle: importance of late postnatal development and potential reciprocal interactions between biosynthesis and degradation. *Front. Endocrinol. (Lausanne).* 4:59 10.3389/fendo.2013.00059 PMC3664331 23750153

[B17] Kanamori K. Ross B. D. (2011). Chronic electrographic seizure reduces glutamine and elevates glutamate in the extracellular fluid of rat brain. *Brain Res.* 1371 180–191. 10.1016/j.brainres.2010.11.064 21111723

[B18] Ke Y. Cohen B. M. Bang J. Y. Yang M. Renshaw P. F. (2000). Assessment of GABA concentration in human brain using two-dimensional proton magnetic resonance spectroscopy. *Psychiatry Res.* 100 169–178. 10.1016/S0925-4927(00)00075-5 11120443

[B19] Kehr J. Ungerstedt U. (1988). Fast HPLC estimation of gamma-aminobutyric acid in microdialysis perfusates: effect of nipecotic and 3-mercaptopropionic acids. *J. Neurochem.* 51 1308–1310. 10.1111/j.1471-4159.1988.tb03101.x 3418350

[B20] Khan R. Gorski W. Garcia C. D. (2011). Nanomolar detection of glutamate at a biosensor based on screen-printed electrodes modified with carbon nanotubes. *Electroanalysis* 23 2357–2363. 10.1002/elan.201100348 22735259 PMC3379819

[B21] Long L. H. Halliwell B. (2011). Artefacts in cell culture: α-ketoglutarate can scavenge hydrogen peroxide generated by ascorbate and epigallocatechin gallate in cell culture media. *Biochem. Biophys. Res. Commun.* 406 20–24. 10.1016/J.BBRC.2011.01.091 21281600

[B22] Mazzei F. Botrè F. Lorenti G. Porcelli F. (1996). Peroxidase based amperometric biosensors for the determination of γ-aminobutyric acid. *Anal. Chim. Acta* 328 41–46. 10.1016/0003-2670(96)00089-X

[B23] Medina-Ceja L. Pardo-Peña K. Morales-Villagrán A. Ortega-Ibarra J. López-Pérez S. (2015). Increase in the extracellular glutamate level during seizures and electrical stimulation determined using a high temporal resolution technique. *BMC Neurosci.* 16:11. 10.1186/s12868-015-0147-5 25887152 PMC4363345

[B24] Monge-Acuña A. A. Fornaguera-Trías J. (2009). A high performance liquid chromatography method with electrochemical detection of gamma-aminobutyric acid, glutamate and glutamine in rat brain homogenates. *J. Neurosci. Methods* 183 176–181. 10.1016/j.jneumeth.2009.06.042 19596377

[B25] Moussawi K. Riegel A. Nair S. Kalivas P. W. (2011). Extracellular glutamate: functional compartments operate in different concentration ranges. *Front. Syst. Neurosci.* 5:94. 10.3389/fnsys.2011.00094 22275885 PMC3254064

[B26] Nath K. A. Ngo E. O. Hebbel R. P. Croatt A. J. Zhou B. Nutter L. M. (1995). Alpha-ketoacids scavenge H2O2 in vitro and in vivo and reduce menadione-induced DNA injury and cytotoxicity. *Am. J. Physiol.* 268 C227–C236. 10.1152/ajpcell.1995.268.1.C227 7840152

[B27] Niwa O. Kurita R. Horiuchi T. Torimitsu K. (1998). Small-volume on-line sensor for continuous measurement of gamma-aminobutyric acid. *Anal. Chem.* 70 89–93. 10.1021/ac970740z 9435468

[B28] Reinhoud N. J. Brouwer H. J. Van Heerwaarden L. M. Korte-Bouws G. A. H. (2013). Analysis of glutamate. GABA, noradrenaline, dopamine, serotonin, and metabolites using microbore UHPLC with electrochemical detection *ACS Chem. Neurosci.* 4 888–894. 10.1021/cn400044s 23642417 PMC3656761

[B29] Robinson D. L. Hermans A. Seipel A. T. Wightman R. M. (2008). Monitoring rapid chemical communication in the brain. *Chem. Rev.* 108 2554–2584. 10.1021/cr068081q 18576692 PMC3110685

[B30] Robinson D. L. Wightman R. M. (2007). “Rapid dopamine release in freely moving rats,” in *Electrochemical Methods for Neuroscience* eds Micheal A. C. Borland L. M. (Boca Raton, FL: CRC Press/Taylor & Francis) 17–34. 21204389

[B31] Robinson M. B. Jackson J. G. (2016). Astroglial glutamate transporters coordinate excitatory signaling and brain energetics. *Neurochem. Int.* 98 56–71. 10.1016/j.neuint.2016.03.014 27013346 PMC4969184

[B32] Rowley H. L. Martin K. F. Marsden C. A. (1995a). Decreased GABA release following tonic-clonic seizures is associated with an increase in extracellular glutamate in rat hippocampus in vivo. *Neuroscience* 68 415–422. 10.1016/0306-4522(95)00159-G 7477952

[B33] Rowley H. L. Martin K. F. Marsden C. A. (1995b). Determination of in vivo amino acid neurotransmitters by high-performance liquid chromatography with o-phthalaldehyde-sulphite derivatisation. *J. Neurosci. Methods* 57 93–99. 10.1016/0165-0270(94)00132-Z 7791370

[B34] Sandberg S. G. Garris P. A. (2010). “Neurochemistry of addiction: monitoring essential neurotransmitters of addiction,” in *Advances in the Neuroscience of Addiction* 2nd Edn eds Kuhn C. M. Koob G. F. (Boca Raton, FL: CRC Press/Taylor & Francis) 120–169. 21656979

[B35] Sekioka N. Kato D. Kurita R. Hirono S. Niwa O. (2008). Improved detection limit for an electrochemical γ-aminobutyric acid sensor based on stable NADPH detection using an electron cyclotron resonance sputtered carbon film electrode. *Sensors Actuat. B Chem.* 129 442–449. 10.1016/j.snb.2007.08.040

[B36] Sethi M. L. (1993). Enzyme inhibition X: colorimetric method for determining gabase activity and its comparison with a spectrophotometric method. *J. Pharm. Biomed. Anal.* 11 613–617. 10.1016/0731-7085(93)80013-Q 8399537

[B37] Smith S. Sharp T. (1994). Measurement of GABA in rat brain microdialysates using o-phthaldialdehyde-sulphite derivatization and high-performance liquid chromatography with electrochemical detection. *J. Chromatogr. B Biomed. Sci. Appl.* 652 228–233. 10.1016/0378-4347(93)E0391-3 8006108

[B38] Song C. Murray T. A. Kimura R. Wakui M. Ellsworth K. Javedan S. P. (2005). Role of (7-nicotinic acetylcholine receptors in tetanic stimulation-induced oscillations in rat hippocampal slices. *Neuropharmacology* 48 869–880. 15829257 10.1016/j.neuropharm.2005.01.003

[B39] Tian J. Lu Y. Zhang H. Chau C. H. Dang H. N. Kaufman D. L. (2004). Gamma-aminobutyric acid inhibits T cell autoimmunity and the development of inflammatory responses in a mouse type 1 diabetes model. *J. Immunol.* 173 5298–5304. 10.4049/jimmunol.173.8.5298 15470076

[B40] Ting Wong C. G. Bottiglieri T. Snead O. C. (2003). GABA, γ-hydroxybutyric acid, and neurological disease. *Ann. Neurol.* 54 S3–S12. 10.1002/ana.10696 12891648

[B41] Tretter L. Adam-Vizi V. (2005). Alpha-ketoglutarate dehydrogenase: a target and generator of oxidative stress. *Philos. Trans. R. Soc. Lond. B Biol. Sci.* 360 2335–2345. 10.1098/rstb.2005.1764 16321804 PMC1569585

[B42] Tseng T. Chang C.-F. Chan W.-C. (2014). Fabrication of implantable. Enzyme-immobilized glutamate sensors for the monitoring of glutamate concentration changes in vitro and in vivo. *Molecules* 19 7341–7355. 10.3390/molecules19067341 24905604 PMC6271204

[B43] Willuhn I. Wanat M. J. Clark J. J. Phillips P. E. M. (2010). Dopamine signaling in the nucleus accumbens of animals self-administering drugs of abuse. *Curr. Top. Behav. Neurosci.* 3 29–71. 10.1007/7854_2009_27 21161749 PMC3766749

[B44] Wilson L. R. Panda S. Schmidt A. C. Sombers L. A. (2017). Selective and mechanically robust sensors for electrochemical measurements of real-time hydrogen peroxide dynamics in vivo. *Anal. Chem.* 90 888–895. 10.1021/acs.analchem.7b03770 29191006 PMC5750107

