# Supplementary material for: A Novel Microbiosensor Microarray for Continuous ex Vivo Monitoring of Gamma-Aminobutyric Acid in Real-Time
Source: Front Neurosci. 2018 Aug 7;12:500. doi: 10.3389/fnins.2018.00500 (PMC6090213; doi:10.3389/fnins.2018.00500)
Supplement: Supplementary file 1 [file Data_Sheet_1.docx]

**A Novel Microbiosensor Microarray for Continuous *Ex Vivo* Monitoring of Gamma-Aminobutyric Acid in Real-Time**

**Supplementary Material**

**Imran Hossain^1^, Chao Tan^1,2^, Phillip T Doughty^2^, Gaurab Dutta^1^, Teresa A Murray^2^, Shabnam Siddiqui^2^, Leonidas Iasemidis^2^ and Prabhu U. Arumugam^1,2^***

**^1^Institute for Micromanufacturing, ^2^Center for Biomedical Engineering Rehabilitation Science, 911 Hergot Ave, Louisiana Tech University, Ruston, Louisiana 71272, USA**

*** Correspondence:**Prabhu U. Arumugam
[parumug@latech.edu](mailto:parumug@latech.edu)

**FIGURE S1**

**
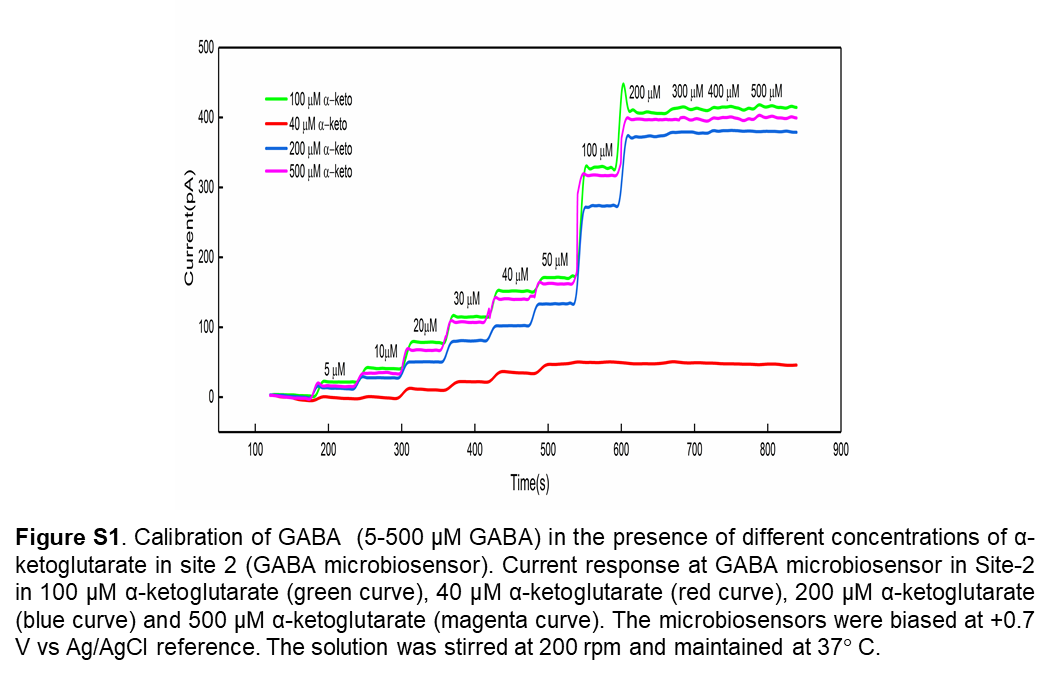
**

**
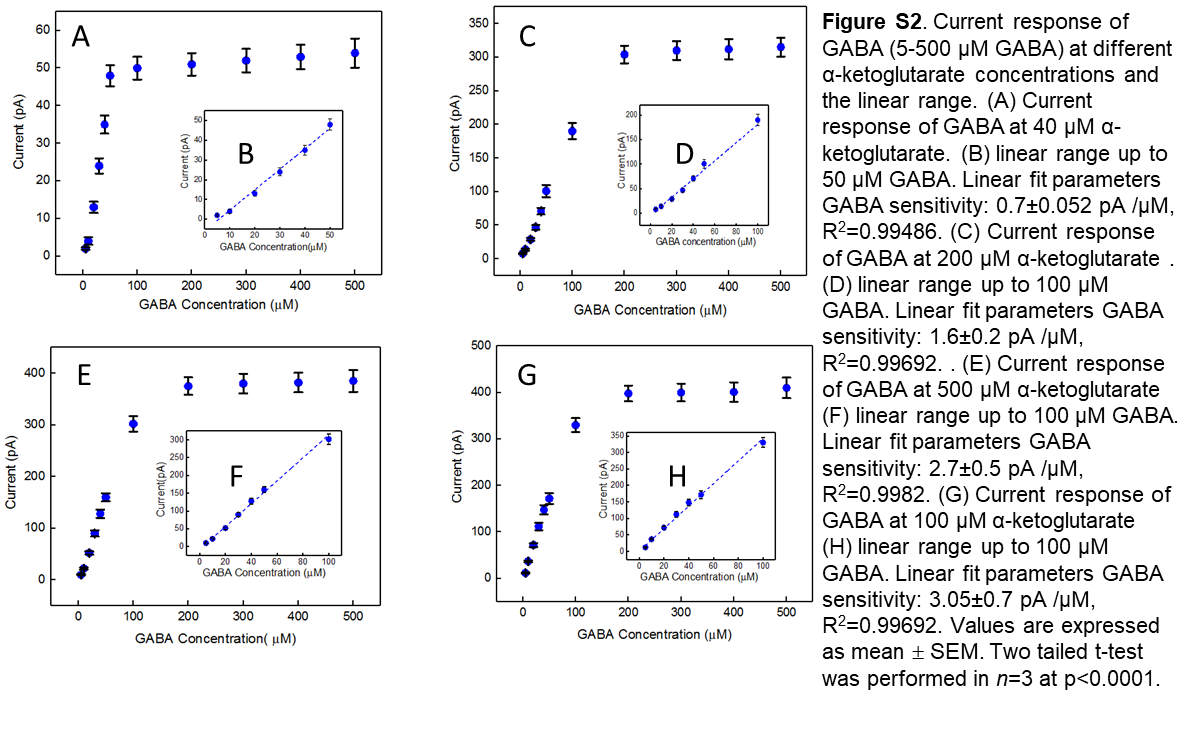
FIGURE S2**

**
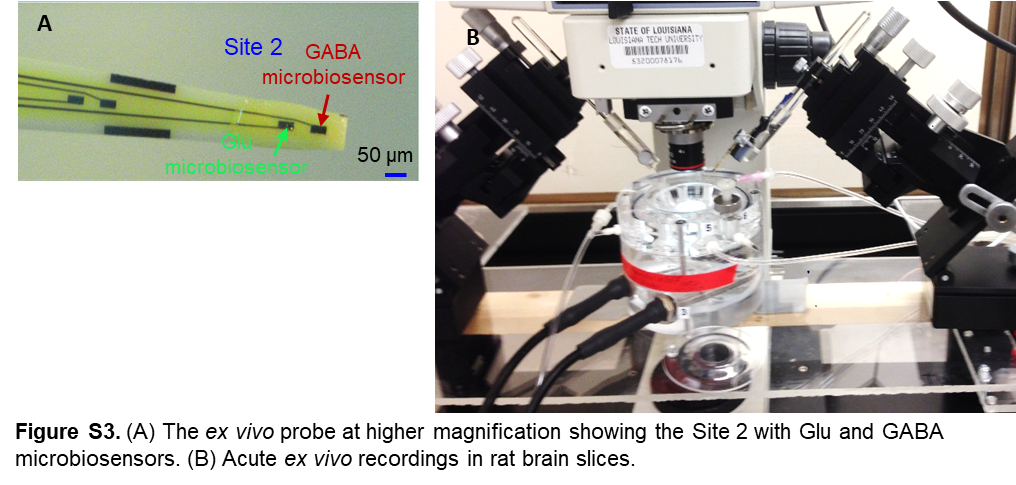
FIGURE S3**
